# Urine albumin dipstick independently predicts cardiovascular and renal outcomes among rural Thai population: a 14-year retrospective cohort study

**DOI:** 10.1186/s12882-020-02215-8

**Published:** 2021-01-08

**Authors:** Noppawit Aiumtrakul, Kitinan Phichedwanichskul, Surapong Saravutthikul, Kamonwan Ottasat, Kesinee Visuthitepkul, Thitinat Jaruthiti, Sarita Jinawong, Kwanchanok Chanthowong, Varot Pengsritong, Nattawinee Horadee, Chotip Jitudomtham, Torpathom Pruekprasert, Thakorn Tawatkiratipol, Tunjira Chokjutha, Panuwat Pongpripoom, Chirayu Wiwatwarapon, Pirawich Sriyarun, Natcha Homrossukhon, Annop Kittithaworn, Wisit Kaewput, Ram Rangsin, Bancha Satirapoj

**Affiliations:** 1 Department of Military and Community Medicine, Phramongkutklao College of Medicine, Bangkok, Thailand; 2 Department of Medicine, Phramongkutklao College of Medicine, Bangkok, Thailand

**Keywords:** Microalbuminuria, Cardiovascular outcomes, Chronic kidney disease

## Abstract

**Background:**

Albuminuria is an established risk marker for both cardiovascular and renal outcomes. In this study, we expected to use portable and inexpensive test strips to detect urine albumin level for risk stratification in cardiovascular and renal outcomes among rural Thai community.

**Objective:**

To evaluate the relationship between urine albumin dipstick and cardiovascular and renal complications in rural Thai population.

**Methods:**

We conducted a retrospective study in 635 rural Thai adults who tested urine albuminuria by using commercial urine albumin dipstick and the Micral-albumin test II strips at baseline. The subjects were divided into normoalbuminuria (albumin < 20 mg/L), microalbuminuria (albumin 20–200 mg/L), or macroalbuminuria (Urine dipstick at least 1+ or albumin > 200 mg/L). We collected data on the incidences of primary composite outcomes including cardiovascular or renal morbidity and mortality. Incident density and cox regression were analyzed to evaluate the association between albuminuria status and primary composite outcome.

**Results:**

During an average 14-year follow-up, 102 primary composite events occurred including 59 (13.1%), 32 (20.6%) and 11 (39.3%) among 452, 155, and 28 subjects with normoalbuminuria, microalbuminuria, and macroalbuminuria, respectively. Incident densities of primary composite outcome were elevated continually according to the degree of albuminuria (9.36, 17.11 and 38.12 per 1000 person-years). Compared with the subjects without albuminuria, subjects with microalbuminuria and macroalbuminuria at baseline had higher risk for primary composite outcome in univariate model. After multivariate analysis was performed, the effect of macroalbuminuria was only persisted with 3.13-fold risk (adjusted HR 3.13; 95% CI 1.40–6.96, *P*= 0.005).

**Conclusion:**

Albuminuria from semi-quantitative methods is an important factor predicting cardiovascular and renal risk among subjects in Thai rural population. Our findings support to also incorporating urine albumin dipstick into assessments of cardiovascular risk in the general population.

## Introduction

The presence of urine albumin reflects endothelial and vascular damage [1]. Albuminuria is an established risk marker for both cardiovascular and renal outcomes [2–9] not only in diabetes patients [10, 11] but also in hypertension [12, 13] and in the general population [14, 15]. Some studies also showed microalbuminuria as an independent predictor for cardiovascular and all-cause mortality [14, 16]. In developed countries, independent association between dipstick proteinuria and an increase in cardiovascular morbidity or all-cause mortality among adult were demonstrated in several studies [17–21]. However, there are limited data about proteinuria and its cardiovascular outcome in developing country [22] where the cost-effective screening test should be performed, especially in primary care setting. In this study, we expected to use portable and inexpensive test strips to detect urine albumin level for risk stratification in cardiovascular and renal outcomes among rural Thai community.

## Materials and methods

### Subjects

**Fig. 1:**
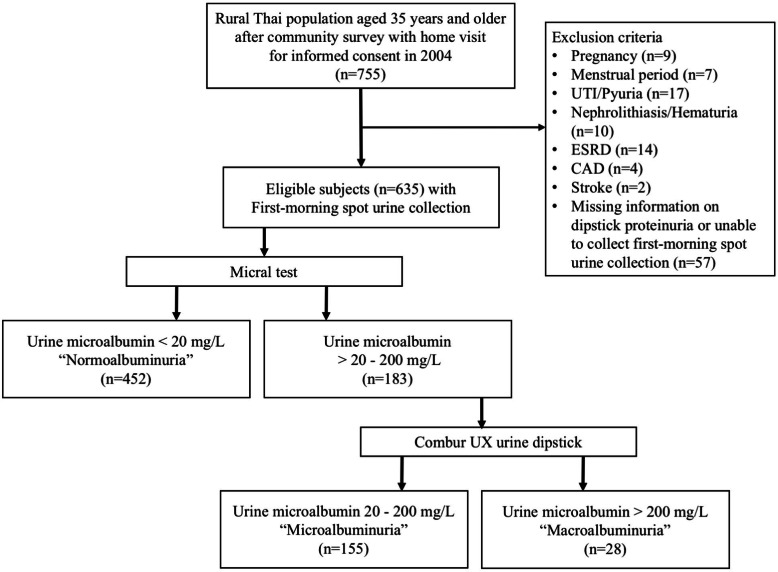
Enrollment flowchart. *UTI* Urinary tract infection, *ESRD* End-stage renal disease, *CAD* Coronary artery disease

A retrospective cohort study was conducted in 755 rural Thai population aged 35 years and older at Moo 15 Baan Nayao Thakadan Subdistrict, Sanamchaikhet District, Chacherngsao Province, Thailand since Febuary 2004. The study protocol was approved by the Institutional Review Board of Royal Thai Army Medical Department’s committee on human research, and written informed consent was obtained from all subjects. Exclusion criteria are pregnancy, having menstrual period, urinary tract infection, pyuria, nephrolithiasis, hematuria, history of coronary artery disease (CAD), history of stroke, end-stage renal disease (ESRD) and subjects who were unable to collect first-spot morning urine or did not have information on dipstick proteinuria. Therefore, 635 subjects were enrolled which consisted of 452, 155 and 28 individuals in Normoalbuminuria, Microalbuminuria and Macroalbuminuria group, respectively (Fig. 1). To study the effect of albuminuria on cardiovascular outcomes, especially mortality, a study by Valmadrid et al. in 2000 was referred [23]. Sample size needed 142, 71 and 36 individuals in Normoalbuminuria, Microalbuminuria and Macroalbuminuria group, respectively.

### Data collection

At baseline, demographic data were obtained using standardized questionnaire including age, gender, weight, height, blood pressure, history of smoking, diabetes, hypertension dyslipidemia and current medication. According to the Seventh Report of the Joint National Committee on Prevention, Detection, Evaluation, and Treatment of High Blood Pressure (JNC 7), hypertensive subjects were defined by office blood pressure measurement with systolic ≥ 140 mmHg or diastolic ≥ 90 mmHg. For medication, ACEI/ARBs included enalapril and losartan, sulfonylurea included glipizide and glibenclamide, beta-blockers included atenolol, propranolol and metoprolol and diuretics included hydrochlorothiazide, furosemide and spinorolactone. These agents were all available medication in primary care centers around the rural community. First-morning spot urine and venipuncture were done for urine dipstick and serum creatinine testing respectively. All urine samples were examined by Micral test to stratify urine albumin group. Subjects who had urine microalbumin less than 20 mg/L defined as “Normoalbuminuria”, for those who had Micral test equal to or over than 20 mg/L will be continually tested by Combur UX urine dipstick. If the samples had urine microalbumin equal to or less than 200 mg/L, the subjects would be defined as “Microalbuminuria”, for those who had over 200 mg/L, they would be defined as “Macroalbuminuria” group. Serum creatinine was tested by enzymatic method. eGFR (in ml/min per 1.73 m2) was calculated using the Chronic Kidney Disease Epidemiology Collaboration (CKD-EPI) [24].

In December 2017, information about cardiovascular events, renal events and all-cause mortality were collected from electronic medical record (EMR) in primary care hospitals nearby, district office and home visit.

### Outcome definition

Primary composite outcome of the study consists of cardiovascular or renal morbidity and mortality. Secondary composite outcome consists of cardiovascular or renal morbidity and all-cause mortality. International Classification of Diseases 10 (ICD-10) codes were identified in the hospital database to collect the outcome from EMR. Cardiovascular event was defined as ICD-I00 to ICD-I52 or ICD-I60 to ICD-I69. Renal event was defined as ICD-N00 to N19 or eGFR lower than 15 mL/min/1.73 m2 or initiation of renal replacement therapy. Information of mortality was obtained from EMR, district office and home visit. The duration between the baseline date and date of first event in each individual was the calculated follow-up time.

### Statistical analysis

Data were analyzed using IBM SPSS 22.0 (SPSS, Chicago, IL, USA). Baseline characteristics of subjects were analyzed using descriptive statistics. Categorical data were presented as number with percentage while continuous data were presented as means with standard deviation. ANOVA test was used to compare continuous variables and Chi-square test was used for categorical variables among albuminuria groups. Incident density was calculated and reported as the number of events per 1000 person-years for both primary and secondary composite outcomes Kaplan-Meier analysis and log-rank test were used for both of the composite outcomes. Cox proportional hazard model for multivariate analysis, adjusting for age, gender, type 2 diabetes, hypertension, eGFR, aspirin, ACEI/ARBs, insulin and metformin at baseline, on both of the composite outcomes was calculated and reported as hazard ratio (HR) with 95% confidence intervals (CI), involving survival time to the first event of any subjects. *P*-values were two-sided and statistical significance was indicated for *p*-value < 0.05.

## Results

We identified 452, 155, and 28 subjects with normoalbuminuria, microalbuminuria and macroalbuminuria, respectively. The subjects in each group had similar characteristic in age, gender, smokers, dyslipidemia patient and the use of beta-blockers, amlodipine, diuretics, sulfonylurea, metformin, simvastatin and gemfibrozil. BMI, blood pressure, hypertensive patient, diabetic patient and the use of ACEI/ARBs, insulin and aspirin were different with higher value as higher level of albuminuria group, except serum creatinine that lower among microalbuminuria than normoalbuminuria group (Table 1).

**Table 1:** Baseline characteristics

	Urine albumin status			***P***-value
	Normoalbuminuria (***n***=452)	Microalbuminuria (***n***=155)	Macroalbuminuria (***n***=28)	
Age (years)	50.48 ± 11.45	51.06 ± 11.31	53.57 ± 12.46	0.359
Male (%)	197 (43.6%)	70 (45.2%)	12 (42.9%)	0.937
BMI (kg/m^2^)	23.54 ± 3.91	24.42 ± 3.93	25.88 ± 4.61	0.016
Systolic BP (mmHg)	121.2 ± 16.45	122.49 ± 16.71	129.38 ± 21.48	0.048
Diastolic BP (mmHg)	77.5 ± 9.19	79.58 ± 10.29	83.46 ± 13.27	0.002
History of Hypertension	112 (24.8%)	50 (32.3%)	14 (50%)	0.005
History of Type2 DM	38 (8.4%)	21 (13.5%)	7 (25%)	0.007
History of Dyslipidemia	39 (8.6%)	10 (6.5%)	4 (14.3%)	0.356
History of smoking (%)	87 (19.2%)	37 (23.9%)	4 (14.3%)	0.340
Serum creatinine (mg/dL)	0.71 ± 0.19	0.68 ± 0.14	0.95 ± 0.48	< 0.001
eGFR (ml/min/1.73m^2^)	91.69 ± 18.85	93.28 ± 16.7	78.8 ± 26.21	0.026
Aspirin (use)	28 (6.2%)	21 (13.5%)	4 (14.3%)	0.009
ACEI/ARBs (use)	45 (10%)	31 (20%)	6 (21.4%)	0.002
Beta-Blockers (use)	24 (5.3%)	9 (5.8%)	1 (3.6%)	0.887
Amlodipine (use)	29 (6.4%)	15 (9.7%)	3 (10.7%)	0.323
Diuretics (use)	46 (10.2%)	16 (10.3%)	5 (17.9%)	0.436
Insulin (use)	7 (1.5%)	7 (4.5%)	2 (7.1%)	0.035
Metformin (use)	30 (6.6%)	18 (11.6%)	4 (14.3%)	0.072
Sulfonylurea (use)	21 (4.6%)	13 (8.4%)	2 (7.1%)	0.208
Simvastatin (use)	45 (10%)	15 (9.7%)	3 (10.7%)	0.985
Gemfibrozil (use)	13 (2.9%)	5 (3.2%)	2 (7.1%)	0.454

Data presents as mean±SD and number with percentage

*BMI* Body mass index, *BP* Blood pressure, *DM* Diabetes mellitus, *eGFR* Estimated glomerular filtration rate, *ACEI/ARBs* Angiotensin-converting enzyme inhibitors/angiotensin II receptor blockers. All variables are at baseline level

The frequency of clinical outcomes and the incident densities of both composite outcomes were increased consecutively according to the degree of albuminuria (Tables 2, 3 and 4).

**Table 2:** Clinical outcomes according to urine albumin status

	Urine albumin status			***P***-value
	Normoalbuminuria	Microalbuminuria	Macroalbuminuria	
Primary Composite Outcomes	59 (13.1%)	32 (20.6%)	11 (39.3%)	< 0.001
Cardiovascular events (ischemic heart disease, HF, PVD, stroke and cardiovascular death)	48 (10.6%)	22 (14.2%)	7 (25%)	0.051
Renal events (ESRD, dialysis and renal death)	19 (4.2%)	8 (5.2%)	7 (25%)	< 0.001
Overall mortality	70 (15.5%)	22 (14.2%)	8 (28.6%)	0.151

Primary composite outcome includes cardiovascular or renal morbidity and mortality. International Classification of Diseases 10 (ICD-10) codes were identified in the hospital database to collect the outcome from EMR. Cardiovascular event was defined as ICD-I00 to ICD-I52 or ICD-I60 to ICD-I69. Renal event was defined as ICD-N00 to N19 or eGFR lower than 15 mL/min/1.73 m2 or initiation of renal replacement therapy

*HF* Heart failure, *PVD* Peripheral vascular disease, *ESRD* End-stage renal disease

**Table 3:** Incident density of primary composite outcome (/1000 person-years)

Primary composite outcome	Person-year (year)	Primary composite outcome (n)	Incident density (/1000 person-years)	95% CI
Normoalbuminuria	5127.92	48	9.36	7.05–12.42
Microalbuminuria	1695.25	29	17.11	11.89–24.62
Macroalbuminuria	288.58	11	38.12	21.11–68.83
**Total**	**7111.75**	**88**	**12.37**	**10.04–15.25**

Primary composite outcome includes cardiovascular or renal morbidity and mortality

**Table 4:** Incident density of secondary composite outcome (/1000 person-years)

Secondary composite outcome	Person-year (year)	Secondary composite outcome (n)	Incident density (/1000 person-years)	95% CI
Normoalbuminuria	5269	109	20.69	17.15–24.96
Microalbuminuria	1733.50	43	24.81	18.40–33.45
Macroalbuminuria	288.58	13	45.05	26.16–77.58
**Total**	**7291.08**	**165**	**22.63**	**19.43–26.36**

Secondary composite outcome includes cardiovascular or renal morbidity and all-cause mortality

Univariate analysis of cox regression model in primary composite outcome, compared with the subjects without albuminuria, subjects with microalbuminuria and macroalbuminuria at baseline had a 1.82-fold and 4.09-fold respectively higher risk for cardiovascular or renal morbidity and mortality (unadjusted HR 1.82; 95% CI 1.15–2.89, *P*= 0.011 and unadjusted HR 4.09; 95% CI 2.21–7.88, *P*= < 0.001). Significant associations were also found in age, gender, type 2 diabetes, hypertension, serum creatinine, eGFR and the use of aspirin, ACEI/ARBs, insulin and metformin as well. In multivariate model, macroalbuminuria, hypertension and aspirin were still significant with 3.13-fold, 2.6-fold and 5.28-fold higher risk respectively (adjusted HR 3.13; 95% CI 1.4–6.96, *P*= 0.005, adjusted HR 2.6; 95% CI 1.35–5.0, *P*= 0.004 and adjusted HR 5.28; 95% CI 2.75–10.14), whereas, metformin significantly reduced risk for 0.29- fold (adjusted HR 3.31; 95% CI 0.09–0.94) after adjusting for age, gender, type 2 diabetes, hypertension, eGFR, aspirin, ACEI/ARBs, insulin and metformin, (Table 5).

**Table 5:** Univariate and multivariate of primary composite outcome

Baseline variables	Univariate		Multivariate	
	Crude HR (95%CI)	***p***-value	Adjusted HR^**a**^ (95%CI)	***p***-value
Normoalbuminuria	Reference	1	Reference	1
Microalbuminuria	1.82 (1.15, 2.89)	0.011	1.4 (0.8, 2.44)	0.235
Macroalbuminuria	4.09 (2.12, 7.88)	< 0.001	3.13 (1.4, 6.96)	0.005
Age (years)	1.04 (1.02, 1.05)	< 0.001	1.02 (1, 1.05)	0.084
Male (%)	1.55 (1.02, 2.36)	0.04	1.26 (0.74, 2.15)	0.397
BMI (kg/m^2^)	1.02 (0.96, 1.08)	0.498		
Systolic BP (mmHg)	1.01 (0.99, 1.02)	0.266		
Diastolic BP (mmHg)	1 (0.98, 1.02)	0.867		
History of Hypertension	3.26 (2.14, 4.95)	< 0.001	2.6 (1.35, 5)	0.004
History of Type2 DM	3.18 (1.95, 5.19)	< 0.001	1.64 (0.48, 5.66)	0.43
History of Dyslipidemia	1.6 (0.85, 3.02)	0.143		
History of smoking (%)	1.33 (0.82, 2.16)	0.249		
Serum creatinine (mg/dL)	3.85 (1.53, 9.68)	0.004		
eGFR (ml/min/1.73m^2^)	0.98 (0.97, 0.99)	< 0.001	0.99 (0.98, 1.01)	0.365
Aspirin (use)	6.73 (4.33, 10.49)	< 0.001	5.28 (2.75, 10.14)	< 0.001
ACEI/ARBs (use)	2.86 (1.79, 4.57)	< 0.001	0.64 (0.32, 1.27)	0.204
Insulin (use)	5.67 (2.84, 11.31)	< 0.001	1.19 (0.42, 3.4)	0.738
Metformin (use)	2.41 (1.36, 4.28)	0.003	0.29 (0.09, 0.94)	0.038

Primary composite outcome includes cardiovascular or renal morbidity and mortality

*BMI* Body mass index, *BP* Blood pressure, *DM* Diabetes mellitus, *eGFR* Estimated glomerular filtration rate, *ACEI/ARBs* Angiotensin-converting enzyme inhibitors/angiotensin II receptor blockers. All variables are at baseline level

^a^Adjusted by age, gender, type 2 diabetes, hypertension, eGFR, Aspirin, ACEI/ARBs, Insulin and Metformin

For secondary composite outcome, univariate cox regression analysis revealed similar trend and significant variables as the previous model, except microalbuminuria. Multivariate analysis with the same adjusting factors showed that age, hypertension and aspirin were significantly associated with the outcome with 1.05-fold, 1.9-fold and 2.08-fold risk respectively. (adjusted HR 1.05; 95% CI 1.02–1.07, *P*= < 0.001, adjusted HR 1.9; 95% CI 1.16–3.11, *P*= 0.011 and adjusted HR 2.08; 95% CI 1.2–3.6, *P*= 0.009) However, macroalbuminuria with 1.92-fold risk might be counted as “clinically” significant factor in multivariate model (adjusted HR 1.92; 95% CI 0.9–4.08, *P*= < 0.09) (Table 6).

**Table 6:** Univariate and multivariate of secondary composite outcome

Baseline variables	Univariate		Multivariate	
	Crude HR (95%CI)	***p***-value	Adjusted HR^**a**^ (95%CI)	***p***-value
Normoalbuminuria	Reference	1	Reference	1
Microalbuminuria	1.2 (0.84, 1.71)	0.312	1.14 (0.75, 1.73)	0.549
Macroalbuminuria	2.23 (1.26, 3.97)	0.006	1.92 (0.9, 4.08)	0.09
Age (years)	1.04 (1.03, 1.06)	< 0.001	1.05 (1.02, 1.07)	< 0.001
Male (%)	1.45 (1.07, 1.97)	0.016	1.21 (0.82, 1.79)	0.335
BMI (kg/m^2^)	1.02 (0.97, 1.07)	0.414		
Systolic BP (mmHg)	1 (0.99, 1.01)	0.44		
Diastolic BP (mmHg)	0.99 (0.98, 1.01)	0.439		
History of Hypertension	2.08 (1.53, 2.84)	< 0.001	1.9 (1.16, 3.11)	0.011
History of Type2 DM	2.12 (1.43, 3.15)	< 0.001	1.59 (0.6, 4.2)	0.346
History of Dyslipidemia	1.08 (0.63, 1.83)	0.786		
History of smoking (%)	1.18 (0.82, 1.7)	0.373		
Serum creatinine (mg/dL)	2.92 (1.35, 6.3)	0.007		
eGFR (ml/min/1.73m^2^)	0.98 (0.97, 0.99)	< 0.001	1 (0.99, 1.01)	0.817
Aspirin (use)	3.21 (2.17, 4.75)	< 0.001	2.08 (1.2, 3.6)	0.009
ACEI/ARBs (use)	1.93 (1.32, 2.81)	0.001	0.93 (0.53, 1.61)	0.786
Insulin (use)	3.42 (1.8, 6.49)	< 0.001	1.13 (0.47, 2.7)	0.785
Metformin (use)	1.8 (1.15, 2.83)	0.01	0.47 (0.18, 1.25)	0.131

Secondary composite outcome includes cardiovascular or renal morbidity and all-cause mortality

*BMI* Body mass index, *BP* Blood pressure, *DM* Diabetes mellitus, *eGFR* Estimated glomerular filtration rate, *ACEI/ARBs* Angiotensin-converting enzyme inhibitors/angiotensin II receptor blockers. All variables are at baseline level

^a^Adjusted by age, gender, type 2 diabetes, hypertension, eGFR, Aspirin, ACEI/ARBs, Insulin and Metformin

**Fig. 2:**
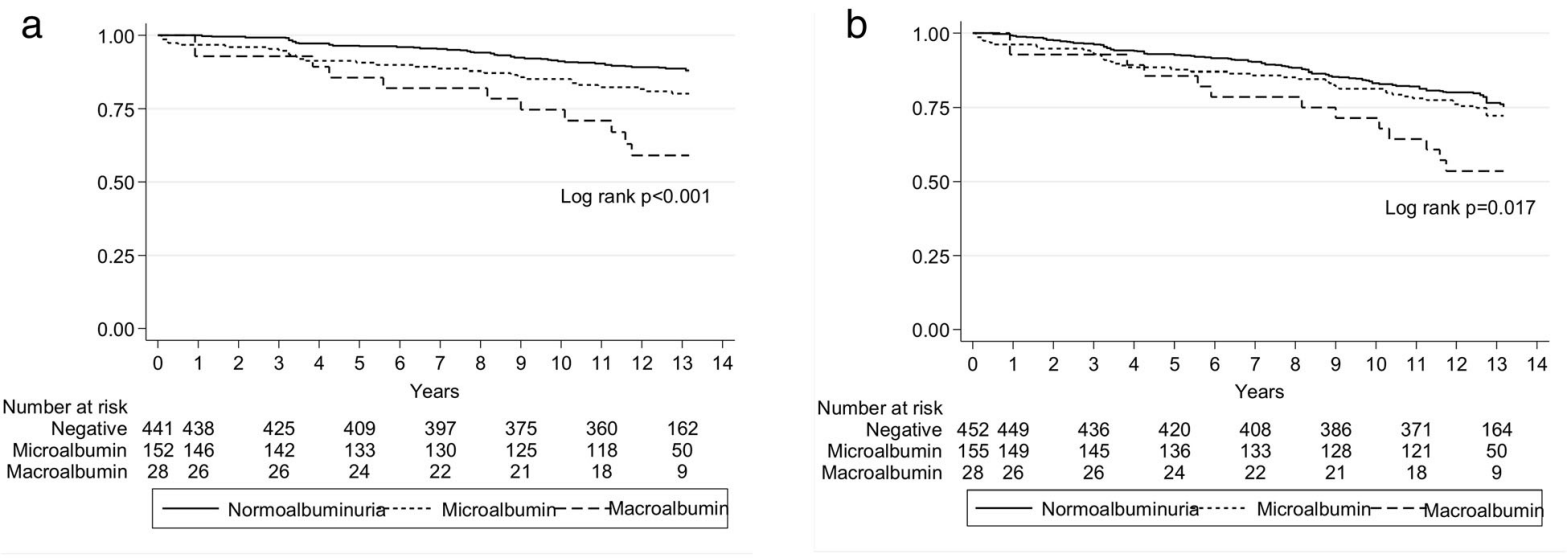
**a** Kaplan-Meier curve for primary composite outcome according to urine albumin status (log rank test < 0.001). **b** Kaplan-Meier curve for secondary composite outcome according to urine albumin status (log rank test = 0.017)

Kaplan–Meier analysis illustrates the consecutively lower survival rate according to higher level of albuminuria for both composite outcomes, with the prominent effect among macroalbuminuria group (log-rank test, *P*=< 0.001, Fig. 2a and log-rank test, *P*=0.017, Fig. 2b).

## Discussion

The 14-year retrospective cohort study showed that urine albumin related to cardiovascular, renal and mortality outcomes. Although, there were other cardiovascular risk factors at baseline, macroalbuminuria still manifested the independent association of cardiovascular and renal complications in primary composite outcome when compared with normoalbuminuria and microalbuminuria.

Results from clinical outcomes, incident density and Kaplan-Meier curve were consistent with previous studies which conducted in diabetic setting. The higher degree of albuminuria had a significant association with higher cardiovascular [23], renal [25] and all-cause mortality [23, 25]. Microalbuminuria had a slightly significant association to the outcome compared with normal urine albumin group while gross albuminuria group had a profound risk.

In this study, multivariate analysis of cox regression model for both composite outcomes did not show microalbuminuria as an independent risk factor. Some prior studies, mostly in diabetes, found that microalbuminuria was not independent risk of cardiovascular [26–31] and mortality [32] outcome as well. On the other hand, significant associations between microalbuminuria and cardiovascular morbidity or mortality [8, 23, 28, 33] and also total mortality [34–44] were reported. For renal outcome, a study by Berhane et al. [25] reported significant association with 2.1 times adjusted hazard ratio (HR) between end-stage renal disease (ESRD) and microalbuminuria when compared with normoalbuminuria. However, most of these studies’ population were diabetic.

Our findings revealed that macroalbuminuria had 3.13-fold higher risk than normoalbuminuria for cardiovascular or renal morbidity and mortality (adjusted HR 3.13; 95% CI 1.4–6.96, *P*= 0.005). It is likely that macroalbuminuria was associated with secondary composite outcome which included all-cause mortality (adjusted HR 1.92; 95% CI 0.9–4.08, *P*= 0.09). Prior studies showed either significant [42, 45–47] or no [27, 33, 40] association between macroalbuminuria and cardiovascular mortality. Whereas, a number of studies found the association between macroalbuminuria and all-cause mortality [34, 36, 37, 43, 48–53]. For renal outcome, several landmark studies discovered albuminuria as risk of ESRD [54–57]. especially macroalbuminuria that had 9.3 times higher of ESRD incidence than normoalbuminuria [25].

Aging, hypertension, aspirin and metformin also revealed the independent relationship to the outcomes. Increased age definitely associated with prevalence and progression of non-communicable disease [58–60], cardiovascular event [61–63] and mortality [64].

Hypertension and diabetes are established risk factors in cardiovascular and renal diseases [65, 66]. We had high proportion of hypertensive subjects at baseline that possibly have an impact on the outcome. Besides, small proportion of type 2 diabetes at baseline could explain why it did not have significant risk to the outcomes. Even though this study does not have baseline fasting plasma glucose, hemoglobinA1c or duration of diabetes to indicate the severity of disease, small amount of insulin use could roughly tell that overall subjects were not poorly controlled diabetes. Therefore, effect of hyperglycemia might not participate much in the outcome’s pathogenesis.

Aspirin had highest magnitude of association in both composite outcomes (adjusted HR 5.28; 95% CI 2.75–10.14) (adjusted HR 2.08; 95% CI 1.2–3.6, *P*= 0.009). Although aspirin is a protective medication for cardiovascular complication [67], this result could be explained by the individuals who took aspirin either for primary or secondary prevention at baseline reflects to their own high cardiovascular risk compared to the person who did not. Furthermore, drug compliance was not assessed in this study which poor compliance is common among patient receiving low-dose aspirin [68].

Some studies suggested that metformin could have benefit in cardiovascular morbidity and mortality [69]. Using metformin at baseline showed risk reduction in primary composite outcome, but not secondary composite outcome.

A strength of the present study was the duration of outcome measurement and ability to detect outcome completely because of small community. Several limitations were noted. The relatively small sample size of our cohort study is the main limitation. There was lacking of urine creatinine concentration and 24-h urine albumin, we cannot evaluate urine albumin-to-creatinine ratio (UACR) or albumin excretion rate to avoid the effect of morning urine hyperconcentration after a night fast. This study was conducted in a primary care hospital in rural Thailand where limited oral anti-hypertensive and diabetic medications were available. Drug compliance and duration of drug use were not evaluated in our retrospective study. Demographic data of subjects who had missing information of dipstick albumin should be measured and compared with the enrolled subjects. Laboratory variables, especially fasting plasma glucose, hemoglobinA1c and lipid profile, were not collected and measured at baseline. Moreover, laboratory data the end of study should be collected as well to assess the change and to be able to create some variable as the outcomes, for example, rapid renal progression. A systemic bias in laboratory measures in urine dipstick and serum creatinine possibly existed. Finally, the generalizability of our findings might be limited by the selection of middle-aged group and subjects living in rural areas of Thailand.

## Conclusion

This study suggests that macroalbuminuria is an independent risk factor for cardiovascular and renal complications, but microalbuminuria is not. Albuminuria from semi-quantitative methods is an important factor predicting cardiovascular and renal risk among subjects in Thai rural population. Our findings support to also incorporating urine albumin dipstick into assessments of cardiovascular risk in the general population.

## Data Availability

The dataset analyzed is available from the corresponding author on reasonable request.
